# Multi-Omics Analysis Provides Novel Insight into Immuno-Physiological Pathways and Development of Thermal Resistance in Rainbow Trout Exposed to Acute Thermal Stress

**DOI:** 10.3390/ijms21239198

**Published:** 2020-12-02

**Authors:** HyeongJin Roh, Ahran Kim, Nameun Kim, Yoonhang Lee, Do-Hyung Kim

**Affiliations:** 1 Department of Aquatic Life Medicine, College of Fisheries Science, Pukyong National University, Busan 48513, Korea; hjroh@pukyong.ac.kr (H.R.); skansl123@naver.com (N.K.); dldbsgkd07@naver.com (Y.L.); 2 Pathology Research Division, National Institute of Fisheries Science, Busan 46083, Korea; ahran110@naver.com

**Keywords:** acute thermal stress, multi-omics, immune disorder, thermal resistance, complement-mediated hemolysis, rainbow trout, transcriptomics, proteomics

## Abstract

In recent years, poikilothermic animals such as fish have increasingly been exposed to stressful high-temperature environments due to global warming. However, systemic changes in fish under thermal stress are not fully understood yet at both the transcriptome and proteome level. Therefore, the objective of this study was to investigate the immuno-physiological responses of fish under extreme thermal stress through integrated multi-omics analysis. Trout were exposed to acute thermal stress by raising water temperature from 15 to 25 °C within 30 min. Head-kidney and plasma samples were collected and used for RNA sequencing and two-dimensional gel electrophoresis. Gene enrichment analysis was performed: differentially expressed genes (DEGs) and differentially expressed proteins (DEPs) were identified to interpret the multi-omics results and identify the relevant biological processes through pathway analysis. Thousands of DEGs and 49 DEPs were identified in fish exposed to thermal stress. Most of these genes and proteins were highly linked to DNA replication, protein processing in the endoplasmic reticulum, cell signaling and structure, glycolysis activation, complement-associated hemolysis, processing of released free hemoglobin, and thrombosis and hypertension/vasoconstriction. Notably, we found that immune disorders mediated by the complement system may trigger hemolysis in thermally stressed fish, which could have serious consequences such as ferroptosis and thrombosis. However, antagonistic activities that decrease cell-free hemoglobin, heme, and iron might be involved in alleviating the side effects of thermally induced immuno-physiological disorders. These factors may represent the major thermal resistance traits that allow fish to overcome extreme thermal stress. Our findings, based on integration of multi-omics data from transcriptomics and proteomics analyses, provide novel insight into the pathogenesis of acute thermal stress and temperature-linked epizootics.

## 1. Introduction

Fish are poikilothermic animals that live in an aquatic environment. Their physiological and metabolic responses are highly correlated with acclimated temperatures [1,2]. Previous studies have reported that the upper thermal limits of aquatic ectotherms often closely correspond to their experienced temperatures [3,4]. Global warming is becoming an ever more serious problem, and the effects of global warming are more severe for ectotherms, including fish, that live in an aquatic environment than animals that live on land [3,5]. Extreme thermal stress is a major abiotic factor that can trigger mortality in teleost fish [6,7,8]. For example, 30% of rahu (*Labeo rohita*) died within two weeks when water temperature was increased by 8.5 °C at a rate of 3 °C day^−1^ [7]. Also, all sea bream (*Sparus aurata*) were killed within one week when water temperature was increased 12 °C at a rate of 6 °C day^−1^ [8]. In light of these trends, the importance of examining the global immuno-physiological characteristics of fish under acute thermal stress cannot be overstated. In general, an elevated water temperature affects immune responses in fish due to thermal stress, leading to temperature-linked epizootics [2,9,10,11,12,13]. Most studies on related topics have used a gradual change rather than an abrupt shift in water temperature to induce stress and observe changes in fish. However, given that contrasting transcriptomic responses might occur in response to chronic vs. acute stress in teleosts [14], identification of systemic biological responses in fish exposed to acute thermal stress would aid in understanding temperature-linked epizootics.

Rainbow trout can grow at temperatures ranging from 8 to 22 °C (optimum temperature: 12–18 °C) [15,16], whereas temperatures above 25 or below 5 °C threaten their survival [17], and they are highly thermal-sensitive species. Thermal stress can trigger tremendous mortality and severe physiological damage [6]. However, the lethal and sub-lethal temperatures of rainbow trout have not been precisely identified. Verhille et al. [18] showed that wild Californian rainbow trout living at extreme latitudes have thermal tolerances that are remarkably higher than those of other *Oncorhynchus mykiss* species from northern latitudes, although they are conspecifics and congenerics. This indicates that thermal tolerance in rainbow trout can change depending on environment, weather, and habitation. Hence, monitoring the physiological and immunological response of the phenotypically high-thermal-resistant strain to high-temperature stress will be of great importance to understanding the mechanism of action of thermal-resistant traits. Such models can be used to identify the molecular biomarkers involved in thermal tolerance in teleost fish [19,20,21].

In recent years, the multi-omics approach (i.e., genomics, transcriptomics, and proteomics) is becoming increasingly popular as it provides a more holistic molecular perspective of a biological system compared to single omics analysis and/or traditional approaches [22]. Many featured studies [23,24] have attempted to use a multi-omics approach to understand the physiological responses of fish (e.g., pufferfish and turbot) under a variety of stressful conditions. These studies have provided broad perspectives and insights. The purpose of this study was to determine the physiological changes of phenotypically thermal-resistant rainbow trout under acute thermal stress and search for novel biological responses. The study aimed to understand the systemic changes and biological signaling mechanisms that underlie complex genetic traits in rainbow trout by integrating global transcriptomic and proteomic profiling of head-kidney and plasma samples using RNA-seq and two-dimensional electrophoresis.

## 2. Results

### 2.1. Fish under Acute Thermal Stress

The rainbow trout used in this study appeared to be phenotypically thermal-tolerant as they all survived for two weeks without showing any signs of disease when the water temperature was increased 10 °C from 15 to 25 °C within 30 min.

### 2.2. Genome-Guided Transcriptome Assembly

Twenty RNA-seq libraries were subjected to paired-end sequencing with a read length of 150 bp using an Illumina NextSeq 500 platform. The highest and lowest transcriptome sizes of the samples were 37,155,214 and 30,713,986 bp, respectively. After eliminating adapter and low-quality sequences, the input reads were mapped to a rainbow trout genome. The mapping rate was 85.50–88.67% (Tables S1–S3). Of 46,585 genes, 30,437 met the following criteria: Fragments per kilobase million (FPKM) ≥ 0.3 and expected read count ≥ 5.

### 2.3. Gene Set Enrichment Analysis (GSEA)

**Figure 1:**
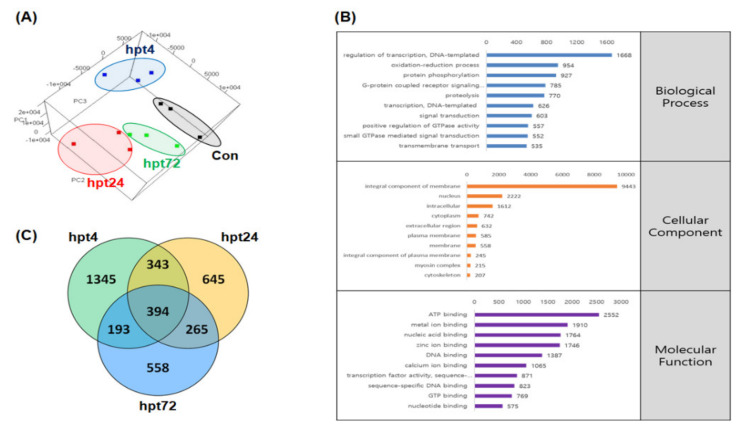
Three-dimensional (3D)-principal component analysis (PCA) of 12 individual transcriptomes in 4 groups. The control, 4, 24, and 72 hpt (hour post thermal stress) groups are shown in black, blue, red, and green, respectively (**A**). Gene ontology (GO) annotation was implemented by Blast2Go and the 20 sub-categories were grouped into three major categories (biological process (BP), cellular component (CC), and molecular function (MF)) (**B**). Venn diagram of number of differentially expressed genes (DEGs) at 4, 24, and 72 hpt (**C**).

**Figure 2:**
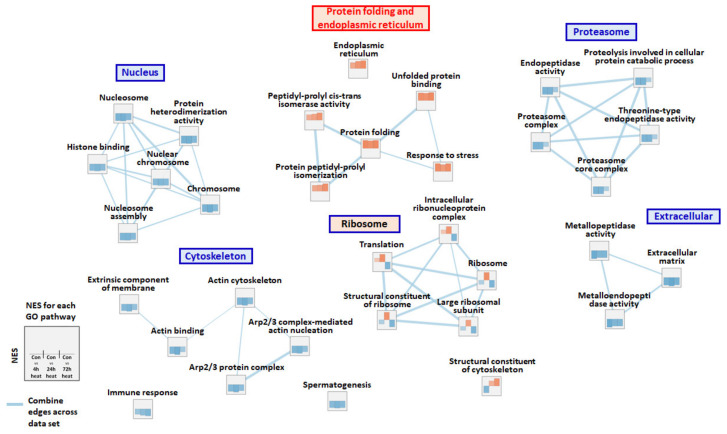
Enrichment map for the shared gene ontology (GO) pathway with *p* < 0.05 and False discovery rate (FDR) < 0.25 during thermal stress. Normalized enrichment scores at 4, 24, and 72 hpt are shown in each node, and the thickness of the light blue edges between the nodes indicates the overlap coefficient across the datasets (4, 24, and 72 hpt).

Based on PCA (principal component analysis), all groups in this study were clustered as shown in Figure 1A. Predicted coding sequences (CDSs) in the trout genome were blasted using Blast2GO (E-value cut-off value 10^−3^) and 30,636 (65.76%) genes were successfully annotated and categorized into three major groups (biological process, BP, cellular component, CC, and molecular function, MF). The size, enrichment score (ES), normalized enrichment score (NES), nominal *p*-value, and false discovery rate (FDR) q-value of each Gene ontology (GO) pathway by comparison with Con (Control) vs. 4 hpt (hour post thermal stress), Con vs. 24 hpt, and Con vs. 72 hpt are shown in Table S4. Regulation of transcription DNA-templated, integral component of membrane, and ATP binding were the most frequently represented sub-categories in BP, CC, and MF, respectively (Figure 1B). In GESA, pathways related to the nucleus, proteasome, cytoskeleton, and extracellular function showed high negative NES values; overall, they were much lower at 4 and 24 hpt than 72 hpt. Additionally, although most pathways relevant to the ribosome had high NES values at 24 hpt, they were downregulated at 72 hpt. However, pathways relevant to protein folding and the endoplasmic reticulum, such as peptidyl-propyl cis-trans isomerase activity, protein peptidyl-prolyl isomerization, protein folding, response to stress, unfolded protein binding, and endoplasmic reticulum showed consistently high NES values (Figure 2).

### 2.4. Differentially Expressed Gene (DEG) Analysis and RNA-Seq Validation

**Figure 3:**
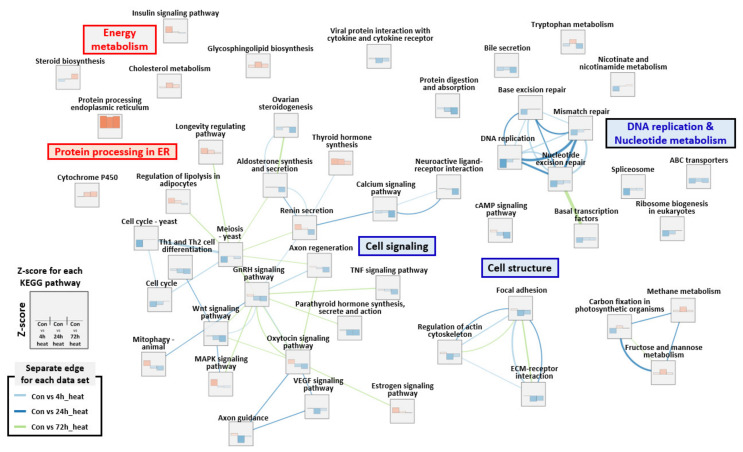
Enrichment map for Kyoto Encyclopedia of Genes and Genomes (KEGG) pathway with Z-score ≥ |2| for at least one time point during thermal stress. Normalized enrichment scores at 4, 24, and 72 hpt are shown in each node. The thickness of the light blue, blue, and light green separated edges between nodes indicates the overlap coefficient at 4, 24, and 72 hpt, respectively.

**Figure 4:**
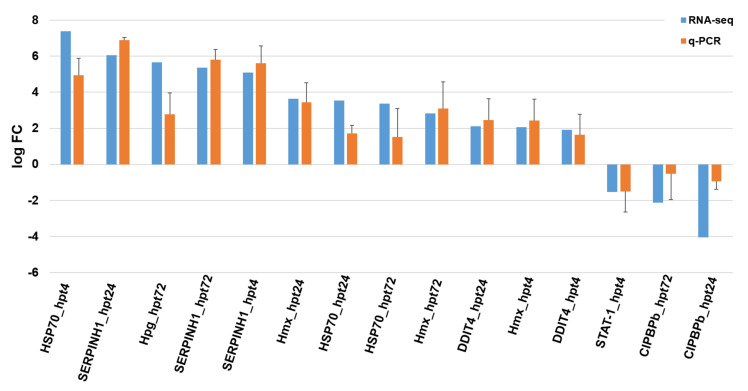
Comparison of gene expression levels between RNA-sequencing and qPCR results.

The DEGs in each group were considered significant when the *p*-value was less than 0.05 compared to control using Edge R: information about these DEGs is available in Table S5. A total of 2274, 1647, and 1411 genes were finally selected as DEGs at 4, 24, and 72 hpt respectively, and 1150 (50.5%), 753 (45.7%), and 676 (47.9%) were successfully annotated in the Kyoto Encyclopedia of Genes and Genomes (KEGG) database (Figure 1C). A total of 3743 genes were significantly altered during the 72 h following thermal stress. At 4, 24, and 72 hpt, the KEGG pathway with the highest Z-scores (4.85, 4.43, and 4.71, respectively) was ko04141 (protein processing in endoplasmic reticulum), which included 54, 56, and 52 DEGs, respectively. Moreover, the KEGG pathways with the lowest Z-scores (−3.59, −2.83, and −2.71) at 4, 24, and 72 hpt were ko03030 (DNA replication), ko04360 (axon guidance), and ko04974 (protein digestion and absorption), respectively. The number of KEGG pathways with │Z-score│ ≥ 2 were 22, 13, and 19 at 4, 24, and 72 hpt respectively, and their Z-scores during thermal stress are shown in Figure 3. During the first 24 h of thermal stress, the Z-scores of pathways relevant to DNA replication and nucleus function, such as DNA replication, base excision repair, mismatch repair, nucleotide excision repair, basal transcription factors, the spliceosome, ABC transporters, etc., decreased with high overlap coefficient, but their Z-scores tended to recover by 72 hpt. On the other hand, the pathways related to cell signaling (e.g., Wingless/int1 (Wnt) signaling pathway, tumor necrosis factor (TNF) signaling pathway, calcium signaling pathway, neuroactive ligand-receptor interaction, cyclic adenosine monophosphate (cAMP) signaling pathway, etc.) and cell structure (focal adhesion, regulation of actin cytoskeleton, extracellular matrix (ECM)-receptor interaction) were not decreased at 4 hpt but had much lower Z-scores at 24 h post-thermal stress (Figure 3). The expression levels of the major functional genes for the featured pathways were validated using quantitative polymerase chain reaction (qPCR), and the expression levels were not significantly different between the qPCR and RNA-seq results (Figure 4).

### 2.5. Serological Analysis

All plasma serological indicators (GOT: glutamic oxaloacetic transaminase, GPT: glutamic pyruvic transaminase, BUN: blood urea nitrogen, ALP: alkaline phosphatase, GLU: glucose, TP: total protein, LDH: lactate dehydrogenase, TCHO: total cholesterol, and Ca: calcium) in rainbow trout in this study are shown in Table 1. Plasma levels of GOT, GPT, and LDH in fish under thermal stress were significantly increased at 72 hpt compared to control fish. However, the plasma level of GLU was significantly increased only at 4 hpt.

### 2.6. Analysis of Plasma Proteome and Differentially Expressed Proteins

**Figure 5:**
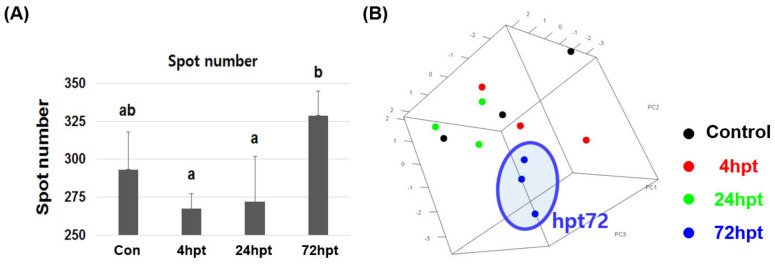
Number of detected spots in the control, 4, 24, and 72 hpt samples (**A**). 3D-PCA based on spot expression among groups (**B**).

**Figure 6:**
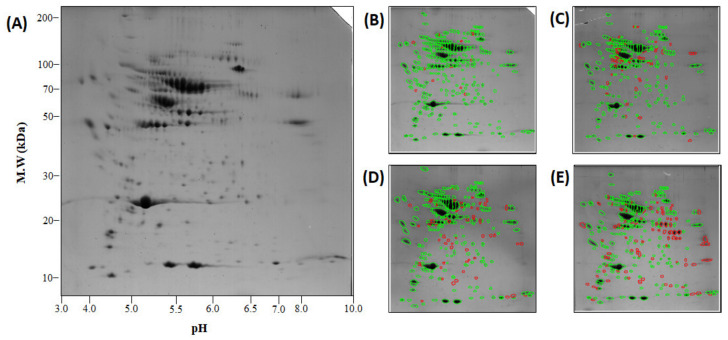
The reference two-dimensional gel electrophoresis (2DE) gel of the plasma in this study. One milligram of protein was separated by isoelectric focusing (IEF) and molecular weight (Mw) (**A**). A representative 2DE gel for control (Con) (**B**), 4 (**C**), 24 (**D**), and 72 (**E**) hpt is shown. All 2DE results and spot ids are available in Table S6. The spots are covered by green and red lines to indicate pair and non-pair spots, respectively.

**Figure 7:**
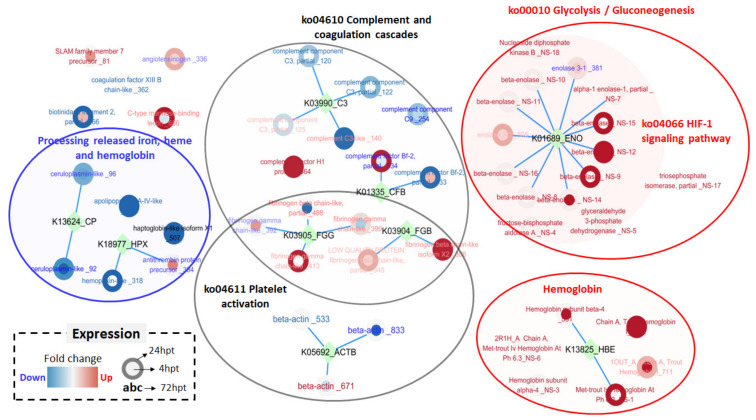
Differentially expressed protein (DEP) expression in the plasma of rainbow trout exposed to thermal stress. The thickness of the red or blue circle, edge, and letters indicates the magnitude of higher or lower expression respectively, at 4, 24, and 72 hpt compared to control. The DEPs were clustered by ko_id with glycolysis/glyconeogenesis, hypoxia-inducible factor 1 (HIF-1) signaling pathway, hemoglobin, platelet activation, complement and coagulation cascades, and processing of released iron, heme, and hemoglobin.

A PCA plot was drawn to show the protein expression patterns in the control and treated groups. The protein expression patterns at 72 hpt clearly differed from those at 4 and 24 hpt (Figure 5). A total of 240–342 spots were observed in each plasma sample after Coomassie blue staining, and the spots at 72 hpt were more numerous than those at the other time points (Figure 6). Likewise, the appearance of non-pair spots at 72 hpt noticeably increased (Figure 6). Fifty-nine, seventy-four, and eighty-eight spots had at least 2 times greater |Mean% of Con volume| at 4, 24, and 72 hpt, respectively (Table S6). Molecular weight (MW), theoretical isoelectric point (pI), sequence coverage (or identity for non-redundant (NR) database), and Mascot score of the 49 DEPs selected in this study are presented in Table S7. The DEPs were divided into five groups based on KEGG annotation: iron processing and release of heme and hemoglobin, complement and coagulation cascades, platelet activation, glycolysis/gluconeogenesis, and hemoglobin (Figure 7). In the complement and coagulation cascades category, the complement components C3 and C9 were significantly downregulated after thermal stress, whereas most complement cofactors, including factor H1 and factor Bf-2, were significantly upregulated. Likewise, many fibrinogen factors (e.g., beta and gamma chain) known to play major roles in platelet activation were observed in plasma. An excessive amount of free hemoglobin was also observed in plasma. Many glycolysis/gluconeogenesis-related proteins (triosephosphate isomerase, fructose-bisphosphate aldolase A, glyceraldehyde-3-phosphate dehydrogenase, enolase, etc.) were also significantly upregulated. However, the levels of plasma proteins related to iron processing and hemolysis (ceruloplasmin, hemopexin, and haptoglobin) were significantly decreased in fish sampled at 4 and/or 24 hpt (Figure 7).

## 3. Discussion

In this study, we profiled the head-kidney transcriptome and plasma proteome of rainbow trout exposed to acute thermal stress using a multi-omics approach. Different methods have been used to induce thermal stress in rainbow trout. In previous studies (Rebl et al. [25] and Huang et al. [13]), water temperature was increased by 1 °C day^−1^ for a week. Gosselin and Anderson [6] found that most juvenile rainbow trout die when the water temperature is increased from 9.5 to 24 °C at a rate of 0.2 °C h^−1^. Kang et al. [26] also showed that 25% of rainbow trout (~350 g) die when water temperature is increased from 18 to 25 °C at 2.5 °C h^−1^. The present study induced acute thermal stress by increasing water temperature from 15 to 25 °C at 20 °C h^−1^, which is a quicker rate of change than those used in previous studies. Nevertheless, no fish used in this study showed any signs of disease or mortality. The rainbow trout exposed to the thermally stressful conditions in this study survived for two weeks, suggesting that they might be phenotypically thermal resistant. However, in previous studies, rainbow trout were killed by exposure to milder heat-shock conditions [6,26].

For the omics analyses, we used head-kidney and plasma samples. Although the head-kidney is an important lymphoid organ in teleost fish that is known to be closely related to temperature-linked epizootics [13,27], RBCs and gills were more commonly used in previous studies [13,25,26,28]. Only one study [13] looked at head-kidney samples of rainbow trout under chronic thermal stress. However, since Huang et al. [13] observed transcriptomics changes under chronic thermal stress (from 18 to 24 °C at a rate of increase of 1 °C per day), still little is known about transcriptomic changes in response to acute thermal stress. Hence, the comparison between acute and chronic thermal stress would contribute to understanding the physiological characteristics of rainbow trout.

In general, two different strategies are used to interpret massive amounts of transcriptome data. One is selecting DEGs based on typical statistic thresholds. The other is gene set enrichment analysis, which identifies significantly enriched or depleted gene sets (e.g., gene ontology terms or pathways) among a list of ranked genes based on differential expression or other statistics [29,30]. Although genes that are not significantly different from those in the control group are culled in DEG analysis, even the selected DEGs can change depending on the statistical method and cut-off value used. In addition, this method may overlook important genes. On the other hand, it is difficult to identify novel pathways and interpret the results in enrichment analysis because all transcriptomes are enriched into well-known gene sets. Therefore, adopting both strategies may be useful in an attempt to compensate for the shortcomings of each one and obtain much more reliable information [31]. In the present study, we mainly focused on the most significantly clustered pathways based on the transcriptomic and proteomic results. In doing so, we identified six types of systemic changes.

### 3.1. DNA Replication Stress

According to a previous study [32], in rainbow trout subjected to thermal stress, genes involved in DNA replication were continuously downregulated, indicating that the fish were under DNA replication stress, defined as transient slowing or stalling of replication forks [32,33]. DNA replication is a fundamental cellular process that ensures accurate duplication of genetic information and cellular proliferation [34]. However, stalled or abnormal termination of DNA replication is generally caused by different types of DNA lesions (incorrect base pairing and damage to the structure of DNA) that can trigger cancer, Fanconi anemia, and bloom syndrome in mammals [32,35,36]. Since most biological pathways based on the gene set networks related to replication stress were downregulated in this study, it is highly likely that acute thermal stress induced considerable DNA replication stress in the fish. When the DEGs were mapped onto KEGG pathways, the DNA replication pathway (ko03030) had the lowest Z-score in the thermally stressed fish sampled at 4 hpt, indicating that acute thermal stress caused an immediate decline in DNA replication. However, this change was not seen in the study by Huang et al. [13], suggesting that chronic thermal stress in fish is unlikely to cause DNA replication stress (Table 2).

### 3.2. Protein Processing in the Endoplasmic Reticulum and Glycolysis

**Figure 8:**
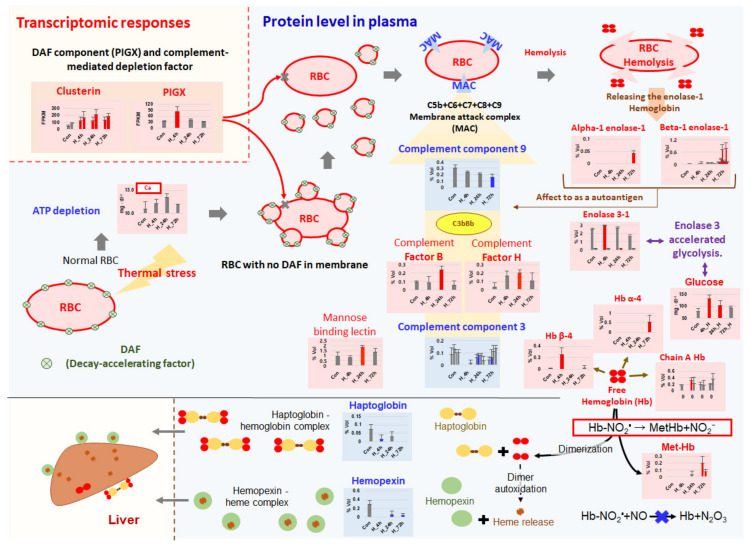
Schematic diagram showing the genes and proteins necessary for processing of complement-mediated hemolysis and removing free hemoglobin and heme during thermal stress.

Protein processing in the endoplasmic reticulum was one of the featured pathways found in the present study. Among all KEGG pathways, ko04141 (protein processing in the endoplasmic reticulum) had the highest Z-score in all samples (i.e., 4, 24, and 72 hpt). Activation of the endoplasmic reticulum may be caused by a rapid increase in the concentration of unfolded and misfolded proteins in fish due to acute thermal stress. In general, thermal stress damages cellular structures, including organelles and the cytoskeleton, leading to re-construction of actin, fiber, and microtubules [37]. Heat stress also has a profound influence on nuclear processes by interrupting RNA splicing. Anormal responses to heat stress could affect large granular depositions and cell swelling, leading to the production of misfolded and/or unfolded proteins [37,38,39]. Cell structure and signaling pathways were downregulated and GOT, GPT, and LDH levels increased, which strongly suggests the presence of substantial cell damage and cellular malfunction in the liver and head-kidney [40,41]. Unassembled and misfolded proteins are usually stored in the endoplasmic reticulum and degraded through ER-associated degradation (ERAD) [42]. Many DEGs belonging to ERAD-related pathways were found to be upregulated in the present study, suggesting ERAD activation in rainbow trout exposed to acute thermal stress. The proteasome is known to be the final destination of the ERAD pathway. However, most DEGs belonging to proteasome-related pathways (e.g., endopeptidase activity, proteolysis involved in the cellular protein catabolic process, threonine-type endopeptidase activity, proteasome core complex, and proteasome complex) were downregulated over time. These conflicting results may be related to proteasomal dysfunction, as previously described [43,44,45]. The proteasome is known to be involved in intracellular protein degradation. However, excessive proteasomal activity creates cytoplasmic inclusion bodies and aggregates, which in turn induce proteasomal inhibitors [45,46,47]. Lee and Goldberg [48] showed that proteasome inhibitors can stimulate the production of heat shock proteins (HSPs). High expression of HSPs was also found in the present study. This may be closely linked to the control of thermal stress in fish. However, changes in protein processing did not seem to differ between acute and chronic stress based on the results of the present study and a previous study [13], although the intensity of gene expression was much greater in fish under acute stress (Table 2). Since the levels of unfolded/misfolded proteins increased rapidly in fish following thermal stress, proteasomal activity might be significantly reduced, followed by the upregulation of endoplasmic reticulum-related gene sets, especially ERAD-related processes. Glycolysis/gluconeogenesis is the fastest method of supplying energy via release of glucose (the major fuel for ATP generation) when a large amount of energy is required during a life-threatening stressful event [49]. In the present study, an increase in the levels of proteins and glucose in the plasma induced upregulation of a glycolysis-related pathway (glycosphingolipid biosynthesis) (Figure 8). Huang et al. [13] also showed that the glycosphingolipid pathway is one of the most significantly enriched pathways in rainbow trout under chronic thermal stress (Table 2).

### 3.3. Complement-Associated Hemolysis

The proteomic analysis in this study revealed a large amount of hemoglobin and met-hemoglobin in the plasma of thermally stressed fish. This is strong evidence of hemolysis during acute thermal stress. Decreases in the levels of the complement components C3 and C9 and increases in the expression of complement cofactor B in the blood may be involved in activation of the complement system on the surface of RBCs. Complement factor B is a cofactor for proteolytic activation of C3b and a catalytic subunit for generation of C3 convertase, while C3 and C9 are major complement components that are immediately utilized [50,51]. In general, erythrocytes can lose some portions of their membranes when producing a large number of vesicles after severe physiological changes such as ATP depletion and an increase in cytosolic Ca^2+^ concentration [52]. Babiker et al. [53] showed that decay-accelerating factor (DAF, a glycosylphosphatidylinositol-anchored membrane protein), one of the major complement inhibitors on the surface of human erythrocytes, falls off with vesicles when the incubation water does not contain ATP but the osmotic pressure is adjusted (285 mosM) or the water is supplemented with 4 µmol L^−1^ Ca^2+^ for one hour [52,53,54,55]. Depletion of DAF and CD59 on the RBC surface allows deposition of the complement system, leading to complement-mediated hemolysis [53,56]. Likewise, in the present study, thermal stress was associated with activation of glycolysis, one of the major responses that counteracts ATP depletion in the host [57], and high Ca^2+^ levels in the plasma. This physiologically unstable condition was directly linked to the loss of many surface components, including complement inhibitors such as CD59 and DAF, on trout RBCs [55,58]. In response to complement-dependent cytotoxicity, the gene expression levels of clusterin and PIGX, known as a complement lysis inhibitor and DAF constituent, were significantly increased in head-kidney samples [59,60]. Likewise, complement factor H1, a key factor known to protect host cells and tissues from damage caused by increased complement activation [61], was also significantly upregulated in thermally stressed fish at 24 hpt. Our results imply that an inability of inhibitors to prevent complement activation on the surface of erythrocytes could trigger complement-mediated auto-immunity linked to hemolysis (Figure 8). Indeed, it is well-known that complement-mediated hemolysis, platelet activation, and thrombosis occur in humans under oxidative stress (e.g., Fibach and Dana [62], Amer et al. [63], Nagababu et al. [64]). Since strong complement activation was not seen in trout under chronic thermal stress [13], the complement-mediated hemolysis observed in this study may be a unique characteristic of fish under acute thermal stress (Table 2).

### 3.4. Removal of Cell-Free Hemoglobin

Cell-free heme/hemoglobin released from hemolysis can trigger tissue damage through severe inflammation and reactive oxygen species stimulation, leading to disorders such as sepsis, sickle cell disease, atherosclerosis, and dysfunction of multiple organs [65,66,67,68,69,70]. Since hemopexin and haptoglobin can bind and dispose of cell-free heme/hemoglobin in the blood, animals have to maintain a certain amount of those molecules to minimize the deleterious effects of iron-containing compound/proteins during hemolysis [70,71]. Excessive concentrations of ferric ions and copper in the plasma released by hemolysis could cause iron-dependent cell death and necrosis, or “ferroptosis” [72,73]. Copper deposition in cells is known to accelerate Parkinson’s disease, Wilson’s disease, and Alzheimer’s disease in humans [74,75,76,77,78]. To cope with excessive ferric and copper ion levels, the host maintains a certain amount of ceruloplasmin to prevent ferroptosis [75,79,80]. The significant reduction in the ceruloplasmin level in the plasma of fish exposed to acute thermal stress seen in the present study might have been caused by influx of a large number of copper ions into the blood, followed by hemolysis. The biochemical reaction between ceruloplasmin and Cu^2+^ usually generates nitric oxide (NO•), and NO• oxidizes hemoglobin to met-hemoglobin in a hypoxic environment [81,82,83]. Accordingly, the met-hemoglobin observed in stressed fish at 72 hpt in the present study may be due to increased nitric oxidation (Figure 8). Given that hemolysis was not seen in response to chronic thermal stress in a previous study [13], the removal of cell-free hemoglobin released from hemolysis and several subsequent steps might be featured biological reactions only in fish under acute thermal stress (Table 2). These kinds of antagonistic activities might be very important thermal resistance traits that protect fish from acute thermal stress.

### 3.5. Thrombosis and Hypertension/Vasoconstriction

**Figure 9:**
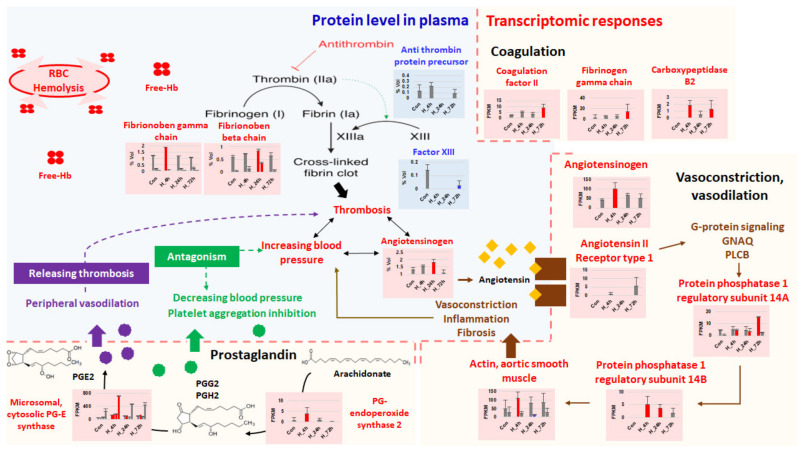
Schematic diagram showing genes and proteins necessary for thrombosis and modulation of vasoconstriction and vasodilation during thermal stress.

In this study, the protein levels of anti-thrombin precursor and coagulation factor XIII B chain decreased in thermally stressed fish, whereas the levels of fibrinogen beta and gamma chains increased significantly. Fibrinogen, a major component of fibrin for blood coagulation, is known to be upregulated under thrombosis, tissue injury, and systemic inflammation [84,85,86,87,88]. Indeed, the transcriptional levels of coagulation pathway-associated genes, such as coagulation factor II, fibrinogen gamma chain, and carboxypeptidase B2, were upregulated in fish under thermal stress. These results indicate that tissue damage and thrombosis are likely to occur in response to blood coagulation in fish exposed to acute thermal stress (Figure 9).

Angiotensinogen is a precursor form of angiotensin that causes vasoconstriction and increases blood pressure. Interestingly, both transcription and protein levels of angiotensinogen were significantly increased in the present study under acute thermal stress. Since angiotensinogen increases platelet sensitivity and blood pressure through the renin-angiotensin system, it is widely known to have a prothrombotic effect [89,90,91]. This indicates that angiotensinogen is promptly produced and secreted from the kidney to the blood. Our results indicate that a high level of angiotensinogen in the plasma of fish under thermal stress can cause vasoconstriction. This finding coincides with the results of previous studies which demonstrated a correlation between thrombosis and hypertension/vasoconstriction in zebrafish as well as humans [92,93,94,95,96]. Interestingly, we also found that prostaglandin G and E were upregulated in thermally stressed fish (Figure 9). Such upregulation may contribute to vasodilation and inhibition of platelet aggregation since prostaglandins are known to regulate blood pressure to alleviate thrombosis-induced hypertension and serve as locally acting vasodilators [97]. It can be argued that these seemingly contradictory results reflect a series of processes that aim to maintain host homeostasis in response to thermal stress. Therefore, acute thermal stress could induce blood coagulation and thrombosis in fish, leading to hypertension and physiological imbalance. Meanwhile, stressed fish can produce prostaglandins to maintain homeostasis.

## 4. Materials and Methods

### 4.1. Acute Thermal Stress

The animal study was reviewed and approved by Animal Research Ethics Committee at Pukyong National University (Approval number: 2017-11; Approval date: 02.05.2017). Rainbow trout (*Oncorhynchus mykiss*) (weight = ~70 g) were obtained from a commercial fish farm (Gyeongsangbuk-do, South Korea; water temperature: 10–19 °C from March to November) and maintained in aerated de-chlorinated freshwater at 15 °C for one week. Acclimated trout were fed daily with commercial dry pellets (1% of body weight). The photoperiod was 12 h of light and 12 h of dark. The health status of the fish was examined immediately upon arrival in the aquaria. To study thermal stress, twenty-five fish were moved to a 125 L tank in which the water temperature was increased from 15 to 25 °C over the course of 30 min to induce acute thermal stress. The temperature was then maintained at 25 °C, and five fish were sampled and sacrificed at each of four time points (0, 4, 24, and 72 h). Zero-hour fish used as a control was sampled at 15 °C, and three among five sampled fish in each time points were randomly selected and used for further analyses. Half of the total volume of water was exchanged every day, and mortality was observed for 2 weeks. The maximum temperature in the tank, 25 °C, is one that salmonids experience during summer in their natural environment [98]. Whole blood and head-kidney samples were obtained from fish treated with an excess of anesthetic agent (MS-222, Sigma). Whole blood was drawn from the caudal vein of sampled fish and anticoagulated with Na-heparin. Plasma was obtained after centrifugation at 12,000 rpm for 10 min at 4 °C. The head-kidney and plasma samples were stored at −80 °C until further analyses.

### 4.2. RNA Extraction and RNA-seq

A total of twelve RNA samples (three replicates at each sampling time point) were extracted using a RNeasy Total RNA Isolation kit (Qiagen, Valencia, CA, USA) according to the manufacturer’s instructions. RNA concentration and quality were determined with a NanoDrop-2000 UV-Vis spectrophotometer (Thermo Fisher Scientific, Wilmington, DE, USA) and a Bioanalyzer 2100 (Agilent Technologies, Santa Clara, CA, USA). RNAs were sheared into short fragments and then double-stranded cDNAs were synthesized and ligated to sequencing adapters using a TruSeqTM RNA Library Prep Kit v2 (Illumina, San Diego, CA, USA) following the manufacturer’s instructions.

### 4.3. Genome-Guided Assembly and Annotation

Adapter sequences and low-quality reads (quality score <20) were removed. High-quality reads were then mapped with published trout genome information (CCAF000000000) using STAR (Ver. STAR-2.5.2a) [99]. The mapped sequences were assembled. Estimated expression abundance was normalized to determine FPKM (fragment per kilobase of transcript sequence per million base pairs) based on RSEM (v. 1.2.31). Principal component analysis (PCA) was performed using the rgl package (v. 0.97.0.) in R software (v. 3.2.5). For functional annotation, the Blast2GO software based on the gene ontology database was used [100]. Annotated genes were categorized by biological process, cellular component, and molecular function [101]. An enrichment map-based GO pathway was drawn to understand the overall gene expression tendency using Enrichment Map App (v. 3.0) in Cytoscape (v. 3.7.2) after gene set enrichment analysis [102,103,104]. The overlap coefficient (OC) was calculated (1). GSEA enrichment score, normalized enrichment score, false discovery rate, and *p*-value of individual gene sets were also calculated [105].

(1) $$\mathrm{Overlap}\;\mathrm{coefficient}\;\left(\mathrm{OC}\right)=\frac{\left|A\;\cap \;B\right|}{\mathrm{Min}\;\left(\left|A\right|,\;\left|B\right|\right)}$$

|A| = number of genetic elements in gene set A.

To simplify the enrichment map, only shared GO pathways with nominal *p*-value <0.05 and FDR <0.25 during thermal stress were included, and their edges were connected by combining edges across the datasets.

### 4.4. Selection of Differentially Expressed Genes and Functional Annotation

To select differentially expressed genes (DEGs) between the control and thermal stress groups (4, 24, and 72 hpt, hours post thermal stress), statistical comparison was done using the edgeR package (v. 3.2.2) in R software (v. 3.2.5) [106]. Every gene with a *p*-value less than 0.05 was considered a DEG. These genes were functionally annotated using the Kyoto Encyclopedia of Genes and Genomes (KEGG) and Swiss-Prot database [107,108]. The number of ko_id and terms from the up- and down-regulated DEGs were used to calculate Z-scores (2). Biologically meaningful KEGG pathways with │Z-score│ ≥ 2 were selected, excluding pathways involved in human disease [109]:

(2) $$Z-score=\frac{{\mathrm{Number}\;\mathrm{of}\;\mathrm{upregulated}\;\mathrm{ko}}_{\mathrm{id}}-{\mathrm{Number}\;\mathrm{of}\;\mathrm{downregulated}\;\mathrm{ko}}_{\mathrm{id}}}{\sqrt{{\mathrm{Number}\;\mathrm{of}\;\mathrm{total}\;\mathrm{ko}}_{\mathrm{id}}}}$$

The KEGG pathways with │Z-score│ ≥ 2 during at least one sampling time point were visualized with an enrichment map, and the overlap coefficients between the pathways at 4, 24, and 72 hpt were separately connected with different edge colors.

### 4.5. Validation of RNA-seq Results Using qPCR

A subset of DEGs highly linked to featured pathways considered to have important functions were validated by comparing the RNA-seq and qPCR results. The expression levels of five DEGs (heat shock protein 70 (HSP70), cold-inducible RNA-binding protein (CIRBPb), serpin peptidase inhibitor1 (SERPINH1), Stat1, and DNA-damage-inducible transcript 4 (DDIT4)) were compared between the control and thermal stress groups at different time points (4, 24, and 72 hpt). Subsequently, 1 μg of total RNA was used for cDNA synthesis with PrimerScript reverse transcriptase (PrimerScript^TM^ RT reagent kit, Takara, Japan) following the manufacturer’s protocol. qPCR reactions were performed using 2 μL of cDNA, 12.5 μL of master premix (qPCR SYBR Green 2× master mix kit, Mbiotech, Inc., Seoul, Korea), 8.5 μL of distilled water, and 2 μL of forward and reverse primers. qPCR was performed under different conditions based on the primers used (Table S8) [110,111,112,113].

### 4.6. Analysis of the Biochemical Factors in Plasma

The following plasma biochemical factors were analyzed using an automated dry chemistry analyzer (FUJI DRI-CHEM 3000) following the manufacturer’s protocol: GOT, GPT, BUN, ALP, GLU, TP, LDH, TCHO, and Ca. Statistically significant differences were verified by one-way analysis of variance (ANOVA) in SPSS (20.0) based on Turkey’s multiple range test.

### 4.7. Two-Dimensional Electrophoresis (2DE)

Protein isolation, quantification, rehydration, isoelectric focusing (IEF), and 2DE were performed based on previously published methods [114,115]. Briefly, plasma samples (three replicates from each sampling time point) were treated with the same volume of 10% trichloroacetic acid, vortexed at high speed, and stored at −20 °C for 2 h. Frozen samples were thawed at room temperature and centrifuged at 14,000 rpm for 20 min at 4 °C. The pellet was suspended in ice-cold acetone (−20 °C) and centrifuged at 14,000 rpm for 20 min. Rehydration solution was used to dissolve the pellet and protein concentration was quantified using the Bradford method. IPG buffer (3–10 NL) and 1 mg of protein were mixed with 350 μL sample buffer. Isoelectric focusing (IEF) was then carried out by steadily increasing the voltage from 100 to 3500 V. 2DE was implemented using a polyacrylamide gel electrophoresis system (Bio-Rad, CA, USA) with the temperature maintained at 10 °C. Electrophoresis was performed at 10 mA for 1.5 h followed by 30 mA for 2 h and 40 mA until blue dye reached the bottom of the gel. Gels were fixed with fixing solution (5% phosphoric acid and 40% methanol in distilled water) and stained with Coomassie G250 for 24 h. Protein spots in each gel were analyzed using an image scanner (GS-710 calibrated imaging densitometer, Bio-Rad, CA, USA). Expression levels of pair- and non-pair spots were normalized by relative spot volume (% volume) and differentially expressed proteins (DEPs) were selected. Significant differences were determined by one-way analysis of variance (ANOVA) in SPSS (20.0) based on Tukey’s multiple range test. A total of 49 DEPs (31 pair and 18 non-pair spots) were selected and peptide identification was performed using liquid chromatography/mass spectrometry/ mass spectrometry (LC-MS/MS; Agilent, Wilmington, DE, USA). The acquired peptide sequences were functionally annotated using Mascot or NR (if no information was available in the Mascot database). To identify DEPs, nano LC-MS/MS analysis was performed with a nano high-performance liquid chromatography system. Product ion spectra were collected in information-dependent acquisition (IDA) mode and analyzed with an Agilent 6530 Accurate-Mass Quadrupole-Time of Flight (Q-TOF) using continuous cycles of one full-scan TOF MS from 300 to 2000 *m*/*z* (1.0 s) plus three product ion scans from 150 to 2000 *m*/*z* (1.5 s each). Precursor *m*/*z* values were selected starting with the most intense ion using a selection quadrupole resolution of 3 Da. The rolling collision energy feature was used to determine collision energy based on precursor value and charge state. The dynamic exclusion time for precursor ion *m*/*z* values was 60 s.

A Mascot algorithm (Matrixscience, London, UK) was used to identify peptide sequences present in a protein sequence database. The Mascot database search criteria were: (1) taxonomy, *Oncorhynchus mykiss*, (2) fixed modification—carboxyamidomethylated at cysteine residues, (3) variable modification—oxidized at methionine residues, (4) maximum number of missed cleavages allowed, 2, (5) MS tolerance,100 ppm, and (6) MS/MS tolerance, 0.1 Da. Only peptides resulting from trypsin digests were considered. A Mascot score ≥ 42 was taken to indicate successful annotation. If there was no adequate information in the Mascot database, the NR (non-redundant) database was used to obtain top hit results.

### 4.8. Data Availability Statement

The datasets analyzed for this study can be found in NCBI Sequence Read Archive (SRA; https://www.ncbi.nlm.nih.gov/sra). The accession numbers are SRX8705284–SRX8705295.

## 5. Conclusions

**Figure 10:**
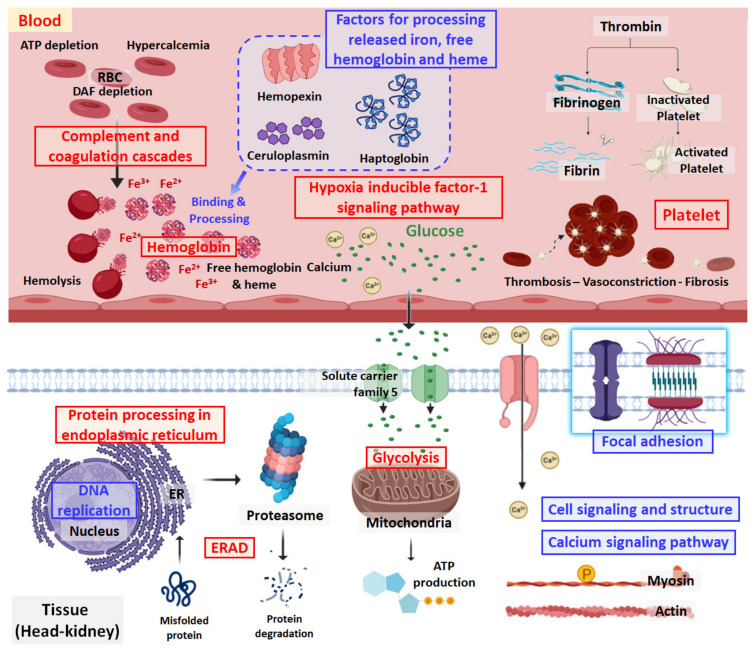
Schematic diagram showing systemic changes in rainbow trout under acute thermal stress. The red and blue boxes indicate featured up- and down-regulated biological responses, respectively. The figure was drawn using Biorender (https://biorender.com/).

To our knowledge, this is the first study demonstrating systemic changes in rainbow trout exposed to acute thermal stress using an integrated multi-omics approach. In the transcriptome analysis, thousands of DEGs were identified in stressed fish at 4, 24, and 72 hpt, indicating that dramatic changes occur in the transcriptome that are greater than those seen in fish under chronic thermal stress [13]. During acute thermal stress, gene sets in pathways related to DNA replication stress, protein processing in the ER, glycolysis, complement-associated hemolysis, processing of released free hemoglobin, and thrombosis were significantly enriched, indicating their importance in this stressful condition. Some biological pathways including protein processing in the endoplasmic reticulum and glycolysis activation of the rate of change of water temperature were shared with chronic thermal stress, while novel-insight responses through multi-omics approaches such as complement-associated hemolysis and subsequent responses were firstly found in this study (Figure 10). Notably, massive hemolysis may have occurred because of disorder of the complement pathway, which seems to be observed under acute thermal stress but not chronic stress. However, since the phenotypically highly thermal-resistant trout used in this study showed fewer side-effects from fatal thermal stress—they did not, for example, show signs of cell death and ferroptosis—they were able to survive at extreme temperatures. This is the first study to explain these physiological characteristics of rainbow trout. The findings can help us understand the immuno-physiological characteristics of rainbow trout, and they will also be useful for identifying and developing thermally tolerant strains of this species with the aim of alleviating the side-effects of thermal stress in the future.

## Figures and Tables

**Table 1:** Changes of serological indicators after acute thermal stress.

	Con	4 hpt	24 hpt	72 hpt
GOT (U·L^−1^)	556 ± 159 ^a^	640 ± 167 ^a^	632 ± 53 ^a^	4811 ± 1213 ^b^
GPT (U·L^−1^)	31 ± 11 ^a^	39 ± 24 ^a^	44 ± 6 ^a^	1128 ± 303 ^b^
ALP (U·dL^−^^1^)	171 ± 135	186 ± 78	300 ± 103	180 ± 13
BUN (mg·dL^−1^)	1.93 ± 0.32	2.30 ± 0.40	2.10 ± 0.46	1.97 ± 0.12
GLU (mg·dL^−1^)	79 ± 13 ^a^	133 ± 17 ^b^	105 ± 19 ^ab^	85 ± 7 ^a^
TCHO (mg·dL^−1^)	268 ± 113	288 ± 71	324 ± 96	230 ± 19
TP (g·dL^−1^)	4.00 ± 0.61	4.40 ± 0.26	4.53 ± 0.32	3.90 ± 0.17
LDH (U·L^−1^)	2012 ± 378 ^a^	3679 ± 234 ^a^	3635 ± 1052 ^a^	12,501 ± 1283 ^b^
Ca (mg·dL^−1^)	10.6 ± 0.9	11.3 ± 0.6	12.1 ± 0.5	11.2 ± 0

GOT: glutamic oxaloacetic transaminase, GPT: glutamic pyruvic transaminase, BUN: blood urea nitrogen, ALP: alkaline phosphatase, GLU: glucose, TP: total protein, LDH: lactate dehydrogenase, TCHO: total cholesterol, and Ca: calcium. Different letters indicated statistically significant differences by Duncan’s multiple range test *(p* < 0.05).

**Table 2:** Comparison of changes of major biological pathways between acute and chronic thermal stress conditions.

Major Biological Pathway	Thermal Stress		
	Acute (In This Study)	Chronic (Huang et al. [13])	
Protein processing in endoplasmic reticulum (ER)	Up	Up	
Glycolysis activation	Up	Up	
Complement associated hemolysis	Up	N.C.	
Processing the hemoglobin	Up	N.C.	
Thrombosis	Up	N.C.	
Hypertension/Vasoconstriction	Up	N.C.	
DNA replication	Down	N.C.	

N.C. = Not changed.

## Supplementary Materials

The following are available online at https://www.mdpi.com/1422-0067/21/23/9198/s1.
