# Supplementary material for: Multi-Omics Analysis Provides Novel Insight into Immuno-Physiological Pathways and Development of Thermal Resistance in Rainbow Trout Exposed to Acute Thermal Stress
Source: Int J Mol Sci. 2020 Dec 2;21(23):9198. doi: 10.3390/ijms21239198 (PMC7731343; doi:10.3390/ijms21239198)
Supplement: Supplementary file 1 [file ijms-21-09198-s001.zip › Table S1.docx]

**Table S1.** The information of total reads and passed reads used in this study.

| **Sample** | **Total read pairs** | **Read1 (Adapter)** | **Read2 (Adapter)** | **Too short** | **Passing filters** |
| --- | --- | --- | --- | --- | --- |
| **Control 1** | 16,875,587 | 4,881,110 (28.9%) | 4,719,267 (28.0%) | 86,306 (0.5%) | 16,789,281 (99.5%) |
| **Control 2** | 17,940,557 | 5,796,494 (32.3%) | 5,516,099 (30.7%) | 66,341 (0.4%) | 17,874,216 (99.6%) |
| **Control 3** | 16,380,138 | 4,164,111 (25.4%) | 4,039,424 (24.7%) | 66,502 (0.4%) | 16,313,636 (99.6%) |
| **4h_heat 1** | 16,508,580 | 4,195,009 (25.4%) | 4,060,758 (24.6%) | 68,163 (0.4%) | 16,440,417 (99.6%) |
| **4h_heat 2** | 16,457,514 | 4,722,402 (28.7%) | 4,557,731 (27.7%) | 62,024 (0.4%) | 16,395,490 (99.6%) |
| **4h_heat 3** | 17,020,816 | 4,474,250 (26.3%) | 4,298,075 (25.3%) | 80,630 (0.5%) | 16,940,186 (99.5%) |
| **24h_heat 1** | 16,554,230 | 4,242,520 (25.6%) | 4,081,112 (24.7%) | 91,977 (0.6%) | 16,462,253 (99.4%) |
| **24h_heat 2** | 17,374,969 | 4,081,295 (23.5%) | 3,964,417 (22.8%) | 57,435 (0.3%) | 17,317,534 (99.7%) |
| **24h_heat 3** | 17,810,177 | 4,571,784 (25.7%) | 4,422,258 (24.8%) | 63,866 (0.4%) | 17,746,311 (99.6%) |
| **72h_heat 1** | 18,577,607 | 4,898,484 (26.4%) | 4,684,939 (25.2%) | 85,624 (0.5%) | 18,491,983 (99.5%) |
| **72h_heat 2** | 15,356,993 | 3,816,877 (24.9%) | 3,616,703 (23.6%) | 79,571 (0.5%) | 15,277,422 (99.5%) |
| **72h_heat 3** | 16,823,397 | 4,081,551 (24.3%) | 3,946,716 (23.5%) | 67,031 (0.4%) | 16,756,366 (99.6%) |
