# Supplementary material for: Multi-Omics Analysis Provides Novel Insight into Immuno-Physiological Pathways and Development of Thermal Resistance in Rainbow Trout Exposed to Acute Thermal Stress
Source: Int J Mol Sci. 2020 Dec 2;21(23):9198. doi: 10.3390/ijms21239198 (PMC7731343; doi:10.3390/ijms21239198)
Supplement: Supplementary file 1 [file ijms-21-09198-s001.zip › Table S2.docx]

**Table S2**. The information of total base pairs and filtered base pairs used in this study.

| **Sample** | **Total base pairs** | **Read 1** | **Read 2** | **Filtered read1** | **Filtered read2** | **Total filtered read (%)** |
| --- | --- | --- | --- | --- | --- | --- |
| **Control 1** | 5,096,427,274 | 2,548,213,637 | 2,548,213,637 | 2,381,525,904 | 2,381,699,701 | 4,763,225,605 bp (93.5%) |
| **Control 2** | 5,418,048,214 | 2,709,024,107 | 2,709,024,107 | 2,512,589,316 | 2,515,737,308 | 5,028,326,624 bp (92.8%) |
| **Control 3** | 4,946,801,676 | 2,473,400,838 | 2,473,400,838 | 2,338,839,794 | 2,335,639,840 | 4,674,479,634 bp (94.5%) |
| **4h_heat 1** | 4,985,591,160 | 2,492,795,580 | 2,492,795,580 | 2,351,667,716 | 2,350,412,379 | 4,702,080,095 bp (94.3%) |
| **4h_heat 2** | 4,970,169,228 | 2,485,084,614 | 2,485,084,614 | 2,324,538,798 | 2,324,705,016 | 4,649,243,814 bp (93.5%) |
| **4h_heat 3** | 5,140,286,432 | 2,570,143,216 | 2,570,143,216 | 2,416,265,575 | 2,415,260,266 | 4,831,525,841 bp (94.0%) |
| **24h_heat 1** | 4,999,377,460 | 2,499,688,730 | 2,499,688,730 | 2,357,114,843 | 2,355,222,206 | 4,712,337,049 bp (94.3%) |
| **24h_heat 2** | 5,247,240,638 | 2,623,620,319 | 2,623,620,319 | 2,490,180,488 | 2,487,643,847 | 4,977,824,335 bp (94.9%) |
| **24h_heat 3** | 5,378,673,454 | 2,689,336,727 | 2,689,336,727 | 2,546,122,298 | 2,541,589,864 | 5,087,712,162 bp (94.6%) |
| **72h_heat 1** | 5,610,437,314 | 2,805,218,657 | 2,805,218,657 | 2,631,535,318 | 2,633,607,451 | 5,265,142,769 bp (93.8%) |
| **72h_heat 2** | 4,637,811,886 | 2,318,905,943 | 2,318,905,943 | 2,191,464,298 | 2,190,044,895 | 4,381,509,193 bp (94.5%) |
| **72h_heat 3** | 5,080,665,894 | 2,540,332,947 | 2,540,332,947 | 2,408,996,332 | 2,405,619,934 | 4,814,616,266 bp (94.8%) |
