# Supplementary material for: Multi-Omics Analysis Provides Novel Insight into Immuno-Physiological Pathways and Development of Thermal Resistance in Rainbow Trout Exposed to Acute Thermal Stress
Source: Int J Mol Sci. 2020 Dec 2;21(23):9198. doi: 10.3390/ijms21239198 (PMC7731343; doi:10.3390/ijms21239198)
Supplement: Supplementary file 1 [file ijms-21-09198-s001.zip › Table S3.docx]

**Table S3.** Total input reads of control and stressed groups of mapping rate of individual samples

| **Sample** | **Number of input reads** | **Uniquely mapped reads** | **Multi hits** | **Unique mapping rate (%)** | **Multi hit rate (%)** | **Mapped reads (%)** |
| --- | --- | --- | --- | --- | --- | --- |
| **Control 1** | 16,736,493 | 12,919,174 | 1,642,248 | 77.19% | 9.81% | 87.00% |
| **Control 2** | 17,645,251 | 13,105,044 | 2,018,262 | 74.27% | 11.44% | 85.71% |
| **Control 3** | 16,279,850 | 12,649,958 | 1,614,436 | 77.70% | 9.92% | 87.62% |
| **4h_heat 1** | 16,391,110 | 12,466,494 | 1,790,363 | 76.06% | 10.92% | 86.98% |
| **4h_heat 2** | 16,331,415 | 12,324,937 | 1,729,847 | 75.47% | 10.59% | 86.06% |
| **4h_heat 3** | 16,890,919 | 13,062,168 | 1,586,840 | 77.33% | 9.39% | 86.72% |
| **24h_heat 1** | 16,421,677 | 12,742,887 | 1,664,982 | 77.60% | 10.14% | 87.74% |
| **24h_heat 2** | 17,273,268 | 13,458,272 | 1,749,233 | 77.91% | 10.13% | 88.04% |
| **24h_heat 3** | 17,621,133 | 12,901,328 | 2,801,774 | 73.22% | 15.90% | 89.12% |
| **72h_heat 1** | 18,365,862 | 13,838,721 | 1,863,692 | 75.35% | 10.15% | 85.50% |
| **72h_heat 2** | 15,238,574 | 11,816,890 | 1,456,788 | 77.55% | 9.56% | 87.11% |
| **72h_heat 3** | 16,710,692 | 13,011,756 | 1,806,353 | 77.86% | 10.81% | 88.67% |
