# Supplementary material for: Multi-Omics Analysis Provides Novel Insight into Immuno-Physiological Pathways and Development of Thermal Resistance in Rainbow Trout Exposed to Acute Thermal Stress
Source: Int J Mol Sci. 2020 Dec 2;21(23):9198. doi: 10.3390/ijms21239198 (PMC7731343; doi:10.3390/ijms21239198)
Supplement: Supplementary file 1 [file ijms-21-09198-s001.zip › Table S8.docx]

**Table S8.** Primer information in this study.

| **Target gene** | **Sequence** | **Reference** |
| --- | --- | --- |
| **HSP70** | F: 5'- GAA GGT GTC CAA TGC AGT CA -3' | **[110]** |
|  | R: 5'- GAT CCT CAG CAC ATT CAG C -3' |  |
| **Incubation:** 95°C for 5 min followed by 40 cycles of 95°C for 20 s, 49°C for 20s | | |
| **SERPINH1** | F: 5'- ATG CAT CGC ACC GGT CTC TAT A -3' | **[25]** |
|  | R: 5'- ATC TTG CCC ATC CAG GTT TCC A |  |
| **Incubation:** 95 °C for 10 min, followed by 40 cycles of 95 °C for 15s, 60 °C for 10s, 72 °C for 20s | | |
| **CIRBPb** | F: 5'- CTT TTT GTG GGT GGC CTG AGC -3' |  |
|  | R: 5'- AGC ATA GCA TCC TTG GCC TCA T -3' |  |
| **Incubation:** 95 °C for 10 min, followed by 40 cycles of 95 °C for 15 s, 60 °C for 10 s, 72 °C for 20 s | | |
| **DDIT4** | F: 5' – CTC TCA GAC TCC GGG TTT GAC TT -3' | **[111]** |
|  | R: 5'- TCA GAA GGG TCT CCT CCA TTG A -3' |  |
| **Incubation:** 95 °C for 30 s, followed by 40 cycles of 95 °C for 5 s, 60 °C for 60 s | | |
| **Stat1** | F: 5'- TTG AGA GCA TCG ACT GGG AAA A -3' | **[112]** |
|  | R: 5'- GGC TAG GAG GTC ATG GAA ACG T -3' |  |
| **Incubation:** 95 °C for 10 min, followed by 40 cycles of 95 °C for 10 s, 60 °C for 1 min | | |
| **Hmx** | F: 5'- AAC GCA GCC TTT GTT TGT GC -3' | In this study |
|  | R: 5'- TGG CAG ATA GGT CAA TGT CCA G -3' |  |
| **Incubation:** 95 °C for 5 min, followed by 40 cycles of 95 °C for 15 s, 64 °C for 20 s | | |
| **Hpg** | F: 5'- TTA GGC ATC ACG CGA CAA TC -3' | In this study |
|  | R: 5'- AGT CTG ATA CGT TCT GGA AGC C -3' |  |
| **Incubation:** 95 °C for 5 min, followed by 40 cycles of 95 °C for 15 s, 64 °C for 20 s | | |
| **Ef-1α** | F: 5'- GAT CCA GAA GGA GGT CAC CA -3' | **[113]** |
|  | R: 5'- TTA CGT TCG ACC TTC CAT CC -3' |  |
| **Incubation:** 95 °C for 5 min, followed by 40 cycles of 95 °C for 15 s, 64 °C for 1 min | | |
